# Construction of a Multifunctional Separator Based on Poly(terephthaloyl-melamine) for the Thermally Safe Regulation of Lithium-Ion Batteries

**DOI:** 10.3390/molecules31081304

**Published:** 2026-04-16

**Authors:** Yiwei Yu, Yongshun Liang, Dan You, Wenhao Yang, Ziyi Zhu, Yingjie Zhang, Linqiang Duan, Xue Li, Yiyong Zhang

**Affiliations:** 1 National and Local Joint Engineering Research Center for Lithium-ion Batteries and Materials Preparation Technology, Key Laboratory of Advanced Battery Materials of Yunnan Province, School of Metallurgical and Energy Engineering, Kunming University of Science and Technology, Kunming 650093, China; 20232202121@stu.kust.edu.cn (Y.Y.);; 2 Yunnan Hongta Plastics Co., Ltd., Yuxi 653100, China; 3 Yuxi Energy New Materials Co., Ltd., Yuxi 653100, China

**Keywords:** lithium-ion battery, separator, poly(terephthaloyl-melamine), thermal shutdown, safety

## Abstract

The poor thermal stability of commercial polyethylene (PE) separators hinders the further application of lithium-ion batteries (LIBs), yet previous modifications struggle to balance between safety and electrochemical performance. This study proposes an interface modification strategy by forming a poly(melamine terephthalamide) (PTM) coating on the PE separator surface, constructing a “thermal–mechanical–electrochemical synergistic barrier”. The PTMs@PE separator achieves synergistic improvements in thermal shutdown behavior, thermal stability, mechanical strength, and electrochemical compatibility by taking advantage of the temperature-sensitive response of the PE separator, the flame-retardants of the rigid conjugated skeleton with the high nitrogen of PTM, and the electrolyte-affinity of its functional groups. Importantly, the principles between the molecular structure of the PTM coating and the thermal behavior is verified. The results demonstrate that PTM participates in the decomposition process of the PE separator and slows down the degradation rate of the PE chain structure, thereby resulting in a wide-temperature-range thermal shutdown temperature. The PTMs@PE effectively reduces the risk of runaway. The PTMs@PE separator achieves outstanding electrochemical compatibility, achieving a capacity retention rate of 99.27% at 2 C for 500 cycles. Notably, the separator shows high potential for scalable fabrication. This work provides a novel material system and technical pathway for developing highly safe and high-performance LIB separators.

## 1. Introduction

The low-carbon energy and electrification of transportation have promoted the application of lithium-ion batteries (LIBs). It is attributed to their core advantages, such as high specific energy, exceptional cycling stability, and reversible electrochemical behavior [1,2,3]. Moreover, the pursuit of an advanced battery has driven the development of battery materials with high voltage and high capacity. Meanwhile, under extreme operating conditions—such as high-temperature environment and fast-charging modes—the risk of thermal runaway has significantly increased. This challenge has become a vital bottleneck restricting the further application of LIBs [4,5,6]. Mechanistically, the poor thermal stability of commercial polyolefin separators (taking polyethylene, PE, as an example) is a key factor for causing thermal runaway, which results from the crystalline characteristics and thermo-oxidative property of PE molecular chains, thereby failing to meet safety requirements under abnormal battery operating conditions [7,8,9].

As the key medium in LIBs for physically separating the cathode and anode, the performance of the separator is co-determined by its microstructure (porosity, pore-size distribution) and intrinsic properties (thermal stability, mechanical property, electrolyte-philicity), which directly governs the safety and electrochemical kinetics of the battery. Commercial polyethylene (PE) separators dominate the current market, owing to their excellent electronic insulation, controllable porous structure, and low cost. However, the PE separator suffers from low heat-shrinkage temperatures of 120–150 °C, arising from the typical linear alkane structure of its molecular chains. When the temperature of the battery rises sharply, due to abuse situations or high environmental temperature, the thermal motion of the PE molecular chains leads to melting of the crystalline regions, resulting in significant thermal shrinkage and eventual rupturing of the separators, which causes direct contact between the electrodes and triggers an internal short-circuit [10,11,12]. Simultaneously, the heat generated by the short-circuit can further initiate chain reactions, such as electrolyte decomposition, gas generation, even combustion, ultimately inducing thermal runaway. To address the core issue, numerous modifications have been developed, including composite modifications with ceramic particles (Al_2_O_3_, SiO_2_), introduction of polymerization with flame-retardant functional groups, and grafting with high-temperature-resistant polymers (polyimide, poly-m-phenylene isophthalamide) [12,13,14,15,16]. The main objective is to construct a thermally stable barrier to suppress shrinkage of the separators at high temperatures. Nevertheless, the existing modification strategies generally suffer from a bottleneck of unbalanced performance. Ceramic particle coatings can enhance thermal stability through physical support, yet the weak interfacial bonding between functional particles and the PE separator makes the coating prone to peeling off, and the hydrophobic nature of the coating weakens electrolyte wettability. Flame-resistant polymer grafting is often accompanied by increased cross-linking density, which decreases the porosity and influences the pore-size distribution of the separator, highly increasing lithium-ion transport resistance. Thermal stable polymer materials, on the other hand, are limited by complex synthesis processes, high costs, and poor compatibility, which hinders its further commercialization. Therefore, developing a separator that can synergistically improve thermal stability, electrochemical behavior, structural compatibility, and industrialization potential has become a critical scientific issue and technological challenge for safe and highly dependable LIBs [8,16,17,18,19,20,21,22,23,24,25,26,27,28,29].

As a typical aromatic heterocyclic polymer, poly(phthaloyl melamine) (PTM) features a conjugated rigid framework formed by alternating benzene and triazine rings, displaying excellent mechanical strength and highly thermal stable property (thermal decomposition temperature > 300 °C). Moreover, the high content of nitrogen in the triazine rings enables excellent thermal stability by releasing inert gases to interrupt combustion reactions. The amino (–NH–) and carbonyl (C=O) groups in the triazine rings can enhance electrolyte adsorption and wettability through hydrogen bonding with the electrolyte solvents [16,22,23,24,25,26,27,28].

**Figure 1:**
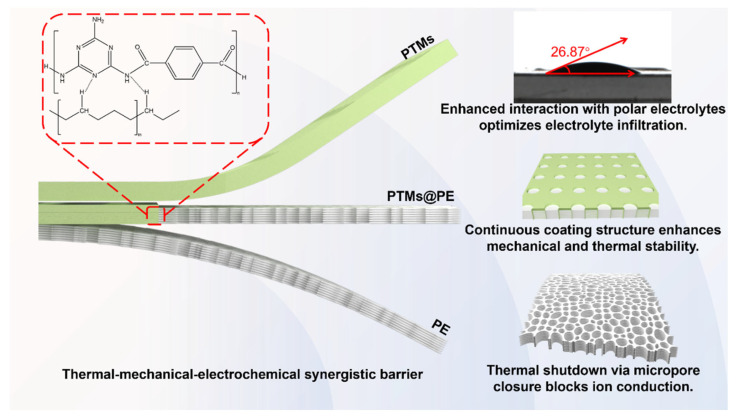
Schematic illustration of the roles of the PTMs@PE separator in the battery.

Herein, we designed and constructed a PTM-coated PE separator (PTMs@PE) through interfacial modification, synergistically combining thermal shutdown capability, thermal stability, and mechanical reinforcement to form an integrated “thermal–mechanical–electrochemical synergistic barrier”. The core advantage of the PTMs@PE separator lies in its temperature-sensitive response enabled by PE, whereby the pore closes near the PE melting point to block ion transport, thereby effectively mitigating short-circuit risks. Meanwhile, the highly rigid heterocyclic skeleton forms a three-dimensional support network with the PE separator, enhancing the mechanical properties and resistance to lithium dendrite penetration. Furthermore, the PTM coating preserves the porous structure while substantially improving electrolyte wettability through hydrogen bonding, thereby enhancing ion transport kinetics. As displayed in Figure 1, the PTM coating exhibits a high thermal decomposition threshold temperature; furthermore, the PTM coating can cross link with a molten PE separator, forming a stable carbon-based and thermal layer. This layer serves as a protective barrier, and exceptionally improves the thermal stability of the PTMs@PE separator. Additionally, the PTM coating forms a robust and continuous layer on the surface of the PE separator. The rigid aromatic and triazine rings within PTM induce a high intrinsic tensile modulus to the separator, significantly improving the overall mechanical strength of the separator. Furthermore, the functional groups (–NH-CO–) in the PTM polymer chains can significantly enhance affinity towards polar electrolytes, leading to better electrolyte infiltration and a more uniform interfacial lithium-ion flux. Consequently, the PTMs@PE separator overcomes the conventional trade-off between safety and electrochemical performance. Specifically, it demonstrates significantly optimized safety and electrochemical characteristics: thermal shrinkage at 150 °C is reduced to below 5% (a marked decrease from >40% for the pristine PE separator), and ionic conductivity remains above 1.2 × 10^−3^ S cm^−1^. In LFP/Li cells with the PTMs@PE separator, a capacity retention of 99.27% is achieved after 500 cycles at 2 C, far surpassing the 97.44% retention of cells with pristine PE separators. Notably, the coating can be uniformly deposited under air atmospheric conditions using a facile approach, demonstrating strong potential for scalable production and providing a pathway toward the industrialization of high-safety separators.

## 2. Results and Discussion

**Figure 2:**
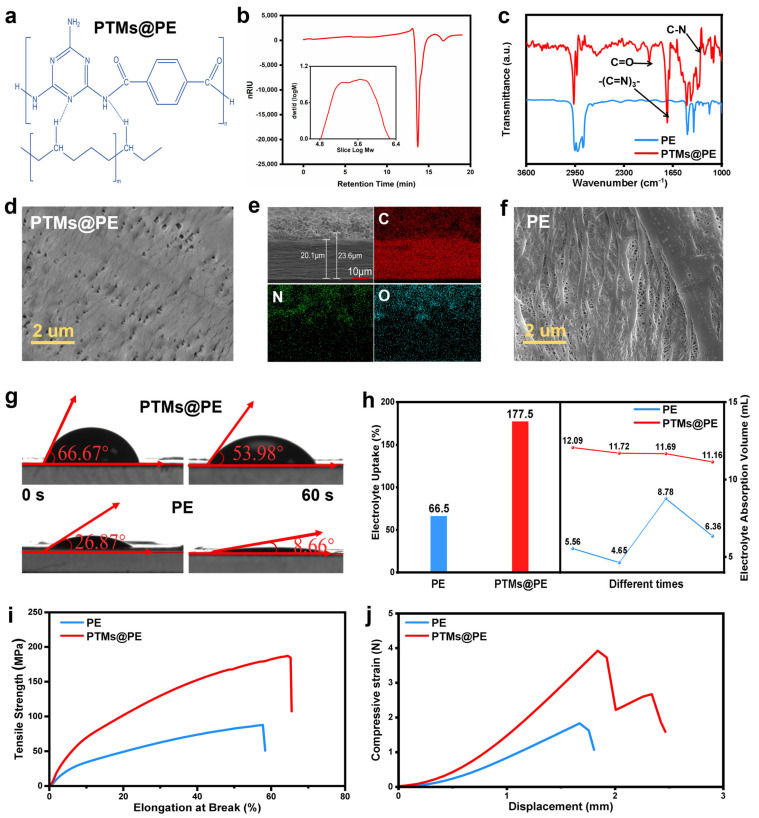
The properties of the PTMs@PE and PE separators: (**a**) the bonding mode of the PTM and PE separators; (**b**) gel permeation chromatography of PTM; (**c**) FT-IR spectra of the PTMs@PE and PE separators; (**d**) surface SEM images of the PTMs@PE separator; (**e**) cross-sectional view and EDS mapping spectra of the PTMs@PE separator; (**f**) subsurface SEM image of the PTMs@PE separator; (**g**) angle contact tests of the PTMs@PE and PE separators; (**h**) the electrolyte uptake of the PTMs@PE and PE separators; (**i**) tensile strength of the PTMs@PE and PE separators; (**j**) nail penetration test of the PTMs@PE and PE separators.

Figure 2a depicts the theoretical interfacial bonding model between PTM and the PE separator. The PTM coating links with the PE separator through hydrogen bonding to construct an integrated structure, attributing for the enhanced structural stability of the PTMs@PE separator. To further confirm the properties of the PTM polymer, gel permeation chromatography (GPC) was conducted (Figure 2b). The GPC results indicate that the average molecular weight (M_n_) of PTM is 257,103 g mol^−1^. Based on a repeating unit molecular weight of 295.5 g mol^−1^, the degree of polymerization (DP) is calculated to be approximately 870. The high molecular weight enables the PTM chains to span over hundreds of nanometers, ensuring the self-assembly of a dense, continuous layer on the PE surface. This nanostructure can endow the PTMs@PE with exceptional thermal stability and mechanical properties. Fourier transform infrared (FT-IR) spectroscopy was employed to further verify the coating mechanism between PTM and the PE separator. (Figure 2c). There are extra characteristic peaks that emerged in the spectra of the PTM@PE separator. The peaks at 1967 cm^−1^ and 1505 cm^−1^ are attributed to a C=O stretching vibration of the amide I band and to an aromatic C=C skeletal vibration, respectively. The peaks at 1273 cm^−1^ are assigned to coupled N–H/C–N vibration. Notably, the typical peak at 1728 cm^−1^ confirms the existence of the triazine ring. All the typical peaks are attributed to the structural features of PTM. Moreover, it can be observed that there is a red shift of the characteristic peaks of the PE separator after the PTM coating. This shift can be attributed to the hydrogen bonding between PTM and PE, which induces a redistribution of electron cloud density. The results provide molecular-level evidence for the tight interfacial integration between PTM and the PE separators, consistent with the mechanism shown in Figure 2a.

Scanning electron microscopy (SEM) tests were performed to observe the microstructure of the separators. The PTM coating forms a uniform and continuous layer on the surface of the PE separator, meanwhile the PTM coating maintains the inherent micropores of the PE separator (Figure 2d,f). Furthermore, the cross-sectional view in Figure 2e exhibits that the thickness of the PTMs@PE separator is 23.6 μm, and the PTM coating can penetrate into the pores of the PE separator with the thickness of 3.2 μm. The results reveal that PTM can construct an integrated PTM–PM@PE–PE structure. This structure is beneficial to maintain the electrolyte transport channels, which then provides a reliable foundation to improve the mechanical strength. The interfacial wettability of the separators was verified by electrolyte uptake and contact angle tests (Figure 2g,h). The results show that the PTMs@PE separator displays an electrolyte uptake of 177.5%, which is 2.7 times higher than that of the PE separator (66.5%), and the electrolyte can spread more rapidly on the PTMs@PE surface with a sharply reduced contact angle, compared with the PE separator. This remarkable enhancement is attributed to the abundant polar functional groups of PTM, such as amide and triazine rings. This establishes a solid foundation for uniform lithium-ion transport and low interfacial impedance, thereby demonstrating that batteries assembled using the PTMs@PE separator exhibit superior electrochemical performance. Additionally, the tensile strength and deformation recovery capability of the separator directly determine the structural stability of the battery during practical usage. If the separator fails to recovers to its original state after long-term cycling, it can readily lead to electrode contact and internal short circuits. The mechanical property of the separator was further investigated. Accordingly, tensile tests were conducted on the separators, and the stress–strain curves are presented in Figure 2i. The tensile strength of the PTMs@PE separator increases steadily with elongation, reaching a peak of approximately 187.2 MPa at an elongation of 65%, followed by a rapid decline due to the failure. By contrast, the tensile strength of the PE separator displays a sharper rise at a low elongation, with a failure elongation of merely 57% and a maximum tensile strength of 87.8 MPa. The overall mechanical performance of the PTMs@PE separator remains outstanding. Furthermore, the infiltrated PTM enhances the toughness and ductility of the separator. Herein, the PTMs@PE separator can withstand a higher elongation and tensile strength. The puncture resistance of the PTMs@PE and PE separators was evaluated by nail penetration tests, and the corresponding results are presented in Figure 2j. The PTMs@PE separator exhibits a puncture force of 3.92 N, which is higher than that of the PE separator (1.83 N). Moreover, it can be discovered that the PTM@PE separator displays less strain than its counterpart at the same applied force, confirming that the PTMs@PE separator can exhibit improved resistance to deformation caused by external stress. It can be concluded that the integrated structure formed between the PTM coating and the PE separator enables the PTMs@PE separator to exhibit greater puncture resistance and a more stable load-bearing performance in practical usage duration. The PTMs@PE separator exhibits outstanding mechanical property and can effectively suppress lithium dendrite penetration during practical battery cycling, thereby mitigating short circuits and improving safety.

**Figure 3:**
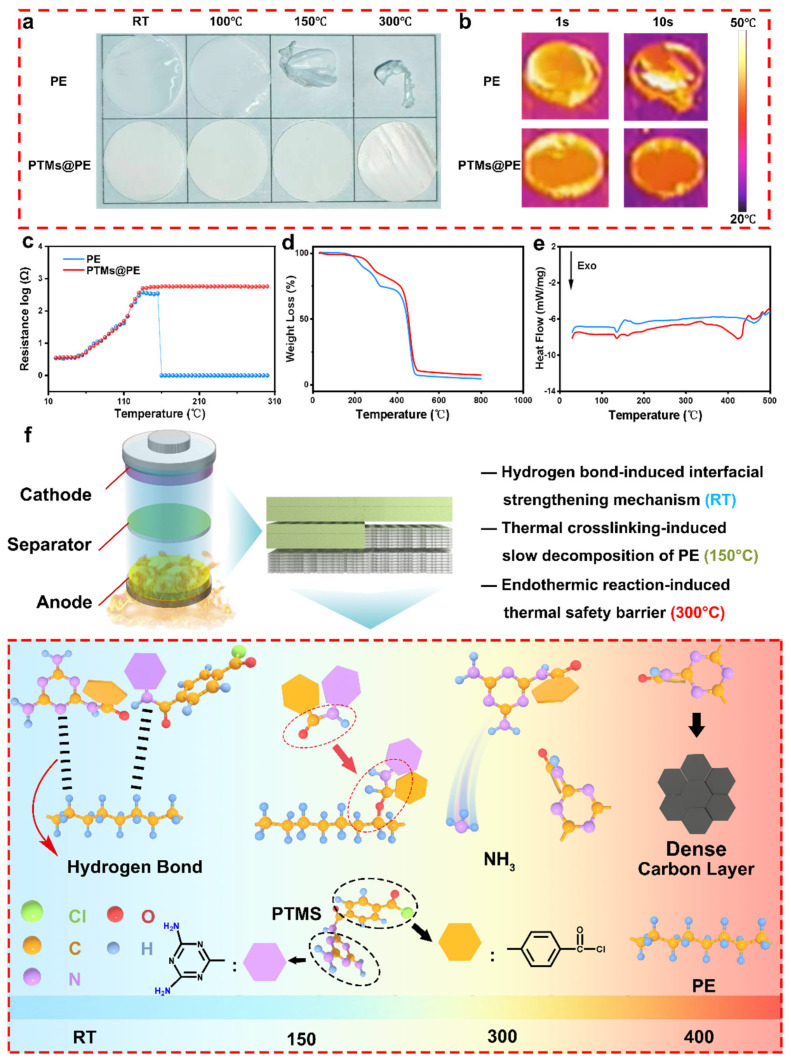
The thermal properties of the PTMs@PE and PE separators: (**a**) optimal images of thermal shrinkage tests of the separators at room temperature, 100 °C, 150 °C, and 300 °C; (**b**) thermal infrared images of the separators; (**c**) the ohmic impedance of the separators at different temperatures; (**d**) thermogravimetry analysis of the separators; (**e**) DSC curves of the separators; (**f**) the working mechanism of the PTMs@PE separator at room temperature, 100 °C, 150 °C, and 300 °C.

The thermal stability of the separators governs the high-temperature safety and commercial application of lithium-ion batteries (LIBs). As shown in Figure 3a, both the PE separator and the PTMs@PE separator maintain a flat state without obvious shrinkage below 100 °C; however, when the temperature rises to 150 °C, the PE separator undergoes severe curling and deformation, while the PTMs@PE separator still maintains its original state. Upon further heating to 300 °C, the PE separator completely melts and fractures, whereas the PTMs@PE separator remains intact, demonstrating an excellent structural retention capability at high temperatures. The thermal infrared test was used to investigate the thermal property of the separators at the heat source of 120 °C (Figure 3b, Video S1 Supporting Information). It can be discovered that the maximum temperature of the PTMs@PE separator is 29.1 °C within 1 s, which is lower than that of the PE separator (33.8 °C). When the heat source keeps working, the PE separator curls up and deforms, whereas, the PTMs@PE separator maintains a complete and stable state. The above results clearly confirm that the PTM coating not only endows the separator with an excellent heat-resistant shrinkage performance but inhibits heat accumulation by enhancing thermal diffusion. In order to accurately verify the thermal behavior of the separators, in situ thermal shutdown tests were performed. It can be seen from Figure 3c that the impedance of both separators increases slowly with the temperature increasing. When the test temperature increases to 120 °C, the two separators display highly increased impedance. The results can be assigned to the motion of the polymer chain which leads to the shrinkage, and finally leads to the uneven Li^+^ pathway. Moreover, the impedance of the PE separator reaches its peak, resulting from the entirely closed pores and blocked Li^+^-conduction pathway. Consequently, the impedance of the PE separator dropped abruptly to nearly zero, suggesting severe separator shrinkage, and finally leading to direct electrode contact and internal short-circuit, whereas the PTMs@PE separator exhibits large impedance even during 200–300 °C. The PTMs@PE separator displays an integrated structure in high temperatures. It can be demonstrated that the thermal shutdown range of the PE separator is only 120–160 °C, whereas the range of the PTMs@PE separator can be extended to 120–300 °C, which significantly enhances the safety of the battery. To deeply understand the mechanism of thermal stability, simultaneous thermal analysis (STA), thermogravimetric analysis (TGA), and differential scanning calorimetry (DSC) were employed to systematically characterize the thermal behaviors of the two separators (Figure 3d,e, Figures S3 and S4). The TGA curves show that both separators exhibit negligible loss in the range of 0–200 °C. The thermal decomposition of the PE separator starts at approximately 186 °C, and the mass loss rate increases sharply after 200 °C until entire decomposition; by contrast, the initial decomposition temperature of the PTMs@PE separator is 244 °C, and the overall mass loss process is much slower. The results demonstrate that the PTMs@PE separator can suppresses the thermal decomposition of the PE separator, thereby endowing the separator with outstanding thermal stability.

A DSC analysis further clarifies the differences in the thermal behavior of the two separators. The pure PE separator exhibits a distinct endothermic melting peak around 120 °C, corresponding to the melting of the PE crystalline region. In the range of 120–160 °C, the PE separator actively closes the pores; however, when the temperature exceeds 160 °C, extensive shrinkage occurs. By contrast, the PTMs@PE separator remains stable in the range of 20–200 °C. The PTMs@PE separator only exhibits a significant endothermic peak at approximately 340 °C, which is attributed to the decomposition or carbonization of the PTM coating itself. This phenomenon indicates that the PTM coating significantly mitigates the decomposition behavior of the PE separator, which can be attributed to the hydrogen bond between the coating and the PE separator.

Combined with electrochemical impedance spectroscopy (EIS) analysis, the PE separator maintains an effective thermal shutdown function only within the narrow window of 120–160 °C, beyond which catastrophic short circuits occur; by contrast, the PTMs@PE separator still maintains a high impedance value at 200–300 °C, demonstrating much superior thermal shutdown durability than the PE separator.

Combining the results of the DSC, the TGA, and the structural evolution characteristics of the separator, as shown in Figure 3f, the excellent thermal stability of the PTMs@PE separator does not originate from a simple increase in the melting point of PE, but is based on the following three-stage synergistic mechanism: in the range from room temperature to 150 °C, the PTM coating forms a stable composite structure with the PE substrate through interfacial interactions, such as hydrogen bonding, which significantly inhibits the segmental motion of PE molecular chains, resulting in the absence of a PE melting endothermic peak in the DSC curve. This is highly consistent with the experimental phenomenon shown in Figure 3a at 150 °C—the PTMs@PE separator maintains a flat morphology without shrinkage or curling, while the pure PE separator undergoes severe deformation, achieving the dual effects of interfacial stabilization and melting inhibition. When the temperature rises to 150–300 °C, exceeding the melting point of PE, the molten PE is physically confined within the pores of the PTM’s three-dimensional network, unable to flow freely or to shrink, and further forms a dense ion-blocking barrier layer. Figure 3e shows that the PTMs@PE separator still maintains an intact outline at 300 °C, while the pure PE separator has completely fractured, confirming the effectiveness of this confinement effect. In the range of 300–400 °C, relying on its high decomposition temperature of 340 °C and slow decomposition kinetics, the PTM coating can maintain its structural framework over a wide temperature range; when the temperature rises to 400 °C, the PTM coating undergoes a carbonization transition and forms a dense carbon layer, providing ultimate flame-retardant protection for the separator. This is the fundamental reason why the PTMs@PE separator can still maintain structural integrity above 300 °C.

**Figure 4:**
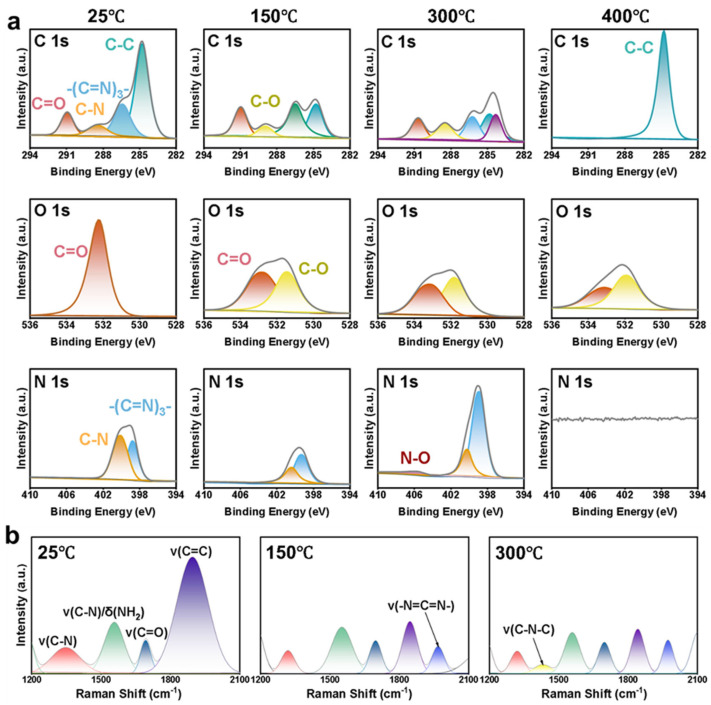
The thermal behavior of the PTMs@PE separator at different temperature and XPS spectra of the PTMs@PE separator: (**a**) C 1 s, O 1 s, N 1 s of the PTMs@PE separator at room temperature, 150 °C, 300 °C, and 400 °C, respectively; (**b**) Raman spectra of the PTMs@PE separator at room temperature, 150 °C, and 300 °C.

To further investigate the thermal stability mechanisms of the PTMs@PE separator, the separator was annealed at three typical temperatures and analyzed by X-ray photoelectron spectroscopy (XPS) and Raman spectroscopy tests, systematically tracking the behavior of the separator under high temperature (Figure 4). The C 1 s spectrum at room temperature can be fitted into four typical peaks. The peak at 284.8 eV is assigned to C–C/C–H bonds, the peak at 286.5 eV corresponds to C–N bonds, the peak at 291.0 eV is attributed to C=O groups, and the peak at 288.5 eV is assigned to the triazine ring of melamine. There is one typical peak in the O 1 s high resolution spectrum, assigned to C=O, and the peaks in the N 1 s spectrum can be fitted into C–N and –(C=N)_3_^−^. When the temperature reaches 150 °C, the C–O bonds are detected. The O 1 s spectrum exhibits an extra peak located at 531.5 eV, which corresponds to C–O. Meanwhile, it can be seen that the intensity of the triazine ring peak (398.9 eV) is higher than the amide peak (400.2 eV). It can be concluded that the partial –CH_2_– in the molten PE separator link with the amide groups to form the C–O bond. The results can clarify the phenomenon of the higher decomposition temperature of PTM, directly suggesting its enhanced thermal stability. Moreover, the XPS analysis reveals that the PE in the PTMs@PE separator is not fully molten and volatized to gases (e.g., CH_2_). Rather, it participates in favorable cross-linking with the PTM coating during decomposition, leading to enhanced thermal stability. When the temperature increases to 300 °C, the intensity of C–O increases in the spectra of C 1 s and O 1 s. The peak of the C=O shifts to lower binding energy (0.2 eV) caused by that the hydrogen bond reorganization results in a higher electron density around the carbonyl oxygen. Furthermore, the FWHM of C=O decreases and the intensity of C–N continues to decrease, suggesting the majority of decomposition of the amide groups. When the temperature reaches 400 °C, there is only a C–C bond detected in the C 1 s spectrum and no characteristic peaks in N 1 s spectrum. This finding is consistent with the decomposition temperatures of the triazine ring (~350 °C) and the amide groups (349–402 °C) in previous research.

In order to investigate the behavior and the structure of the PTMs@PE separator under a practical runway situation, Raman spectroscopy tests were further conducted to verify the above mechanism on the PTMs@PE separator at room temperature, 150 °C, and 300 °C, as shown in Figure 4b. At room temperature, the PTM coating exhibits a highly ordered structure with an I_D_/I_G_ ratio of 0.71 and a full width at half maximum (FWHM) of the G band of only 58 cm^−1^. When the temperature is increased to 150 °C, the G band broadens to 67 cm^−1^, the I_D_/I_G_ ratio increases to 0.83, and a new peak appears at 1968 cm^−1^, corresponding to the coupling vibration between –CONH– and –CH_2_– in the molten PE. This result indicates that there are interfacial cross-linking reactions between PTM and PE to form a rigid network structure, which is consistent with the formation of C–O bonds detected by XPS. It is worth noting that an imine/cyclization characteristic peak also appears at 1895 cm^−1^, confirming the rearrangement of amide groups at high temperatures. When the temperature rises to 300 °C, the I_D_/I_G_ ratio increases significantly to 1.12, the G band shifts to ~1590 cm^−1^, and the intensity of the peaks at 1968 cm^−1^ and 1895 cm^−1^ tend to be equivalent. This indicates that, after the entire melting and decomposition of PE, it forms a further deep cross-link and aromatizes with PTM, forming highly active carbonized microcrystalline domains. This pre-carbonized structure provides a framework for complete carbonization at 400 °C, eventually forming a dense flame-retardant carbon layer. The mechanism revealed by Raman spectroscopy explains the fundamental reason why the PTMs@PE separator can still maintain structural integrity above 300 °C.

Based on the comprehensive analysis above, the thermal behavior of the PTMs@PE separator exhibits multi-stage synergistic characteristics: at 150 °C, PE begins to soften and melt, and PTM forms C–O cross-links with PE through amide groups, initiating the construction of a rigid network and achieving the thermal shutdown function; at 300 °C, PE decomposes completely, the cross-linking reaction proceeds in depth, and a pre-carbonized structure is formed to maintain ion blocking capability and mechanical integrity; at 400 °C, the organic framework is completely converted to generate a dense carbon protective layer, exerting the effect of an ultimate flame-retardant barrier. This progressive mechanism enables the separator to maintain dimensional integrity even under extreme high temperatures, fundamentally expanding the thermal safety boundary of lithium-ion batteries.

**Figure 5:**
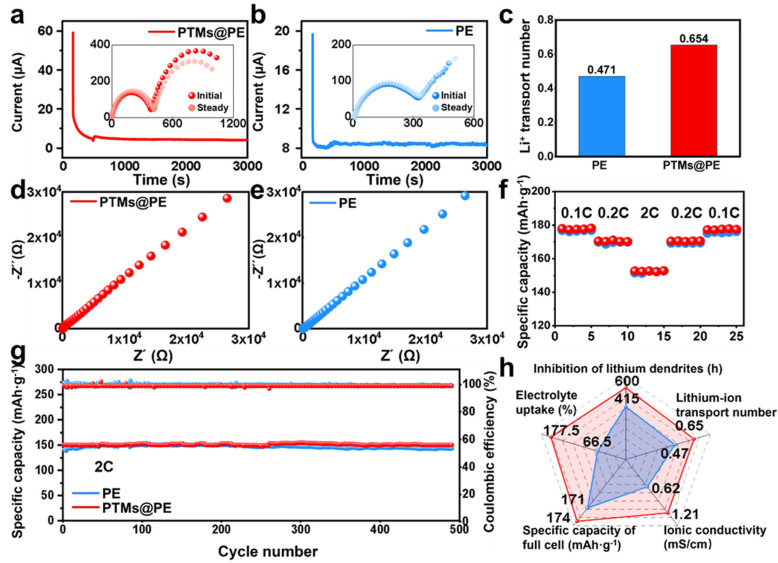
The electrochemical performance of the separators: (**a**) chronoamperometry curves of the PTMs@PE separator and (**b**) the PE separator; (**c**) a comparison of the Li^+^ transference number of the PE and PTMs@PE separators; (**d**) Nyquist curves of the PTM@PE separator and (**e**) the PE separator; (**f**) the rate performance of the PTMs@PE and PE separators; (**g**) electrochemical performance of the PTMs@PE and PE separators at 2 C; (**h**) comparison of the properties of the PTMs@PE and PE separators.

To evaluate the practical application value of the PTMs@PE separator, its electrochemical performance was systematically tested and compared with that of the commercial PE separator. The test results show that the Li^+^ transference number ($t_{Li}^{+}$) of the PTMs@PE separator is 0.65, which is significantly higher than 0.47 of the PE separator (Figure 5a,b). This improvement originates from the selective coordination effect of the abundant amide groups (–CONH–) and triazine rings (−C_3_N_3_−) in the PTM coating on Li^+^: the carbonyl oxygen (C=O) and amino nitrogen (N–H) in the amide bonds, as well as the nitrogen-rich structure of the triazine rings, can form coordination complexes with Li^+^. By immobilizing anions (PF_6_^−^) and promoting Li^+^ dissociation, the lithium-ion transference number is effectively improved. Meanwhile, the PTM coating exhibits excellent wettability to the electrolyte, ensuring sufficient wetting of the electrode/separator interface and reducing the interfacial resistance.

Ionic conductivity tests further confirm the advantage of the PTMs@PE separator (Figure 5d,e). The ionic conductivity of the PTMs@PE separator reaches 1.21 × 10^−3^ S cm^−1^, which is more outstanding than that of the PE separator (6.2 × 10^−4^ S cm^−1^). This enhancement can be attributed to multiple synergistic mechanisms as follows: (1) the amide bonds have a strong dipole moment, which increases the effective dielectric constant of the polymer matrix, weakens the electrostatic interaction between Li^+^ and PF_6_^−^, and promotes lithium salt dissociation; (2) the –(C=N)_3_– structure of the triazine rings and N–H groups form a dynamic hydrogen bond network, providing continuous hopping sites for Li^+^ and reducing the activation energy of ion migration; (3) the conjugated rigidity of the amide bonds in the PTM molecular chains endows the framework with structural stability, while the moderate flexibility of the C–N bonds enhances the segmental motion ability and accelerates ion coupled diffusion; (4) the specific coordination between C=O and Li^+^ further weakens the ion-pair interaction, synergistically improving the ion transport efficiency.

To verify the practical performance of the separators, LiFePO_4_/Li half-cells were assembled for rate performance and cycle stability tests (Figure 5f,g). The rate test results show (Figure 5f) that the cells based on the PTMs@PE separator exhibit a higher specific capacity and excellent capacity reversibility over a wide rate range from 0.1 C to 2 C. The reversible capacity of the PTMs@PE-based cell is 150.1 mAh g^−1^ at 2 C, while that of the PE-based cell is only 147.3 mAh g^−1^; when the rate returns to 0.1 °C, the capacity retention rate of the PTMs@PE-based cell reaches 99.9%, which is significantly superior to the 99.2% of the PE-based cell. This excellent rate performance benefits from the high lithium-ion transference number and ionic conductivity of the PTMs@PE separator, which ensures efficient Li^+^ transport during rapid charge–discharge processes. The galvanostatic charge–discharge (GCD) curves at 2 C (Figure S2) reveal that the cell with the PTMs@PE separator delivers a higher specific capacity compared to that employing the pristine PE separator. Furthermore, long-term cycle stability tests (Figure 5g) indicate that the reversible specific capacity of the PTMs@PE-based cell remains at 151.78 mAh g^−1^ after 500 cycles at 2 C, with a capacity retention rate as high as 99.27% and an average Coulombic efficiency of 99.76%; while, the capacity of the PE-based cell decays to 147.2 mAh g^−1^, with a capacity retention rate of 97.44% and a Coulombic efficiency of 99.21%. The excellent cycle stability of the PTMs@PE separator is attributed to its high lithium-ion transference number that inhibits concentration polarization, good electrolyte wettability that maintains a stable interface, and the firm combination between the coating and the PE separator that avoids structural pulverization during long-term cycling. The comprehensive performance comparison (Figure 5h, Table S1 Supporting Information) clearly demonstrates that the PTMs@PE separator achieves significant overall improvement compared with the commercial PE separator, such as thermal stability (shrinkage rate < 5% vs. >40% at 150 °C), electrolyte wettability (contact angle 66.67° vs. 26.87°), lithium-ion transference number (0.65 vs. 0.47), ionic conductivity (1.21 × 10^−3^ vs. 6.2 × 10^−4^ S cm^−1^), and cycle stability (capacity retention rate 99.27% vs. 97.44% after 500 cycles). Moreover, the shutdown temperature of the PTMs@PE separator is superior to the developed separator in the latest five years (Figure S1 Supporting Information) [16,27,28,29,30,31,32,33,34,35,36,37,38,39,40,41,42,43,44,45]. This synergistic optimization of safety and electrochemical compatibility enables the PTMs@PE separator to meet the requirements of practical applications for energy density and power density while ensuring a high safety, showing broad application value in the new energy market.

## 3. Experimental Section

### 3.1. Materials and Synthesis Method

Melamine (99%, Aladdin, Shanghai, China), Terephthaloyl chloride (99%, Aladdin, Shanghai, China), Dimethyl sulfoxide, (DMSO, 99%, Aladdin, Shanghai, China), N-Methy-l-2-pyrrolidone (NMP, 99.5 wt%, Aladdin, Shanghai, China), polyvinylidene fluoride (PVDF, 99.5 wt%, Aladdin, Shanghai, China), LiFePO_4_ cathode (99.5%, BTR New Material Group Co., Ltd., Shenzhen, China).

Synthesis of the PTMs@PE separator: Melamine and terephthaloyl chloride were added to DMSO at a molar ratio of 2:3, then heated at 120 °C for 4 h to obtain the PTM solution. Consequently, the PTM solution was coated on the commercial PE separator to form a 20 μm layer. And the gained coated separator was kept at −0.8 MPa in a vacuum oven and heated at 60 °C for 24 h until the DMSO completely volatilized, finally the PTMs@PE separator was obtained.

The LiFePO_4_ material, super P, and the PVDF were mixed and grinded at the weight ratio of 8:1:1 to form a uniform slurry, then the slurry was coated on the Al foil. The coated foil was then placed in a vacuum drying oven and dried at 60 degrees Celsius. Then, it was cut into disks with a diameter of 12 mm. The active material mass was calculated using the method of “total electrode mass minus current collector mass”.

The batteries were assembled into a CR 2016-coin cell with a lithium foil (99.9% purity, with a thickness of 1.2 mm, a diameter of 15.6 mm), and the PE or PTMs@PE separator in an Ar glovebox (Etelux Lab2000, Etelux, Beijing, China, O_2_ < 0.01 ppm, H_2_O < 0.01 ppm), with 1M LiPF6 in DMC/EC/EMC = 1:1:1 Vol%.

### 3.2. Electrochemical Characterization

An electrochemical impedance spectroscopy (EIS) test and ion conductivity test were performed on an electrochemical workstation (Autolab, Metrohm, Herisau, Switzerland). The Stainless Steel SS/separator/SS cell pairs were assembled and tested on the Autolab electrochemical workstation with a scanning frequency range of 10^5^ Hz–0.1 Hz. The ion conductivity of the separators was calculated according to the following equation:

$$\sigma =\frac{L}{R\times S}$$

where *σ* is the ionic conductivity, *d* refers to the thickness of the separator, and *R_b_* and *S* refer to the bulk resistance and the area of the SS, respectively.

The Li transference number ($t_{Li}^{+}$) of the different separators was tested with a symmetric cell of Li/separators/Li. The $t_{Li}^{+}$ was calculated according to the following equation:

$$t_{Li}^{+}=\frac{I_{s}(∆V-I_{0}R_{0})}{I_{0}(∆V-I_{S}R_{S})}$$

where $t_{Li}^{+}$ represents the transference number, ∆V is the potential applied on the cell, *I_s_* and *I*_0_ refer to the currents at the steady state and the initial state, respectively, and $R_{S}$ and $R_{0}$ are the steady-state and initial-state resistance, respectively.

### 3.3. Characterization

Scanning electron microscopy (TESCAN GA3, TESCAN, Brno, Czech Republic) was conducted on the separators to observe the morphology. The thermal stability of the materials was tested by a thermogravimetric analysis (TGA, STA 449 F3, NETZSCH, Selb, Germany) and differential scanning calorimetry (DSC, DSC 200 F3, NETZSCH Germany) at the range of 0~1000 °C with the ratio of 5 °C/min. The coating effect between the PTM and the PE separator were investigated by infrared spectrometer (Bruker Vertex 70, Bruker, Brunswick, Germany). The mechanical properties were studied by a stress–stain test and a puncture force test by a universal testing machine.

The Fourier transform infrared spectroscopy (FTIR) spectra were acquired on a Bruker Vertex 70 (Bruker, Germany) over the range of 4000–400 cm^−1^, under the mode of ATR in a dry atmosphere, with background correction applied before data collection. An XPS analysis was carried out on a Thermo Scientific K-Alpha (Thermo Fisher, Waltham, MA, USA) spectrometer (Al Kα source, 1486.6 eV).

Mechanical properties of the PTMs@PE and PE separators were measured using an INSTRON 3343 universal testing machine (Instron, Norwood, MA, USA). Raman spectra were collected on a LabRAM Odyssey micro-Raman spectrometer (HORIBA, Villeurbanne, France). Electrochemical impedance spectroscopy (EIS) was performed on a CHI 1040C electrochemical workstation (Chenhua Instruments, Shanghai, China). A gel permeation chromatography (GPC) analysis was conducted using an Agilent 1260 Infinity II HPLC system (Agilent, Santa Clara, CA, USA). Contact angles were measured using an OCA 20 contact angle goniometer (DataPhysics Instruments, Filderstadt, Germany).

X-ray photoelectron spectroscopy (XPS) tests and Raman tests were conducted on the PTMs@PE separator and the annealed PTMs@PE separators after thermal treatment at 150 °C, 300 °C, and 400 °C for 30 min to check the flame retardant mechanism. Given that the separator sharply decomposes at 400 °C, the solid residue fails meet the test conditions of the Raman test; therefore, no characterization was performed on the PTMs@PE separator at this temperature.

## 4. Conclusions

In summary, this study reports a molecular-level modification strategy to construct a uniform and dense PTM coating on commercial PE separators. Taking advantages of the temperature-responsive property of the PE separator and the intrinsic molecular features of PTM—rich polar functional groups, rigid aromatic heterocyclic backbone, and high thermal decomposition temperature—the designed PTMs@PE separator accomplishes a comprehensive breakthrough in electrochemical compatibility, mechanical robustness, and thermal safety. The polar functional groups (amide and triazine rings) enhance electrolyte wettability via hydrogen-bonding interactions, ensuring efficient lithium-ion transport kinetics. Furthermore, the PTM coating establishes a robust and cross-linking network with the PE separator, significantly improving thermal dimensional stability and mechanical strength, thereby reinforcing resistance to lithium dendrite penetration. Notably, the PTMs@PE separator features a precisely engineered thermal shutdown function: it induces pore closure at 120 °C to terminate ion transport and electrochemical reactions, while simultaneously forming internal cross-linking reactions between PTM and the PE separator across a broad temperature range (120–300 °C) that absorb thermal energy, effectively mitigating heat accumulation and preventing short-circuit risks. Importantly, the fabrication process is straightforward, environmentally compatible, and readily scalable for industrial production. Meanwhile, the coating can be uniformly deposited via a simple dip-coating process, demonstrating significant potential for scalable manufacturing and providing a feasible pathway for the industrialization of high-safety separators.

## Data Availability

Data will be made available upon request.

## Supplementary Materials

The following supporting information can be downloaded at: https://www.mdpi.com/article/10.3390/molecules31081304/s1, Table S1: comparison of the properties of PTMs@PE, PE separator; Figure S1: the shutdown temperature of PTMs@PE separator superior to the developed separator in latest five years; Figure S2: GCD curves of PE and PTMs@PE separators; Figure S3: STA curves of PTMs@PE separator; Figure S4: STA curves of PE separator; Video S1: the thermal infrared test video of the separators at the heat source of 120 °C.
