# Supplementary material for: Construction of a Multifunctional Separator Based on Poly(terephthaloyl-melamine) for the Thermally Safe Regulation of Lithium-Ion Batteries
Source: Molecules. 2026 Apr 16;31(8):1304. doi: 10.3390/molecules31081304 (PMC13119427; doi:10.3390/molecules31081304)
Supplement: Supplementary file 1 [file molecules-31-01304-s001.zip › Supplementing Information.pdf]

Table S1 comparison of the properties of PTMs@PE, PE separators.

	shrinkage rate at 150℃(%)	contact angle (°)	lithium-ion transference number	ionic conductivity (mS/cm)	Specific capacity (mAh·g-1)	capacity retention rate (after 500 cycles at 2C)(mAh·g-1)	Electrolyte uptake (%)	Inhibition of lithium dendrites (h)
PE	40	66.67	0.47	1.21	171	97.44	66.5	415
PTMs@PE	5	26.87	0.66	0.62	174	99.27	177.5	600

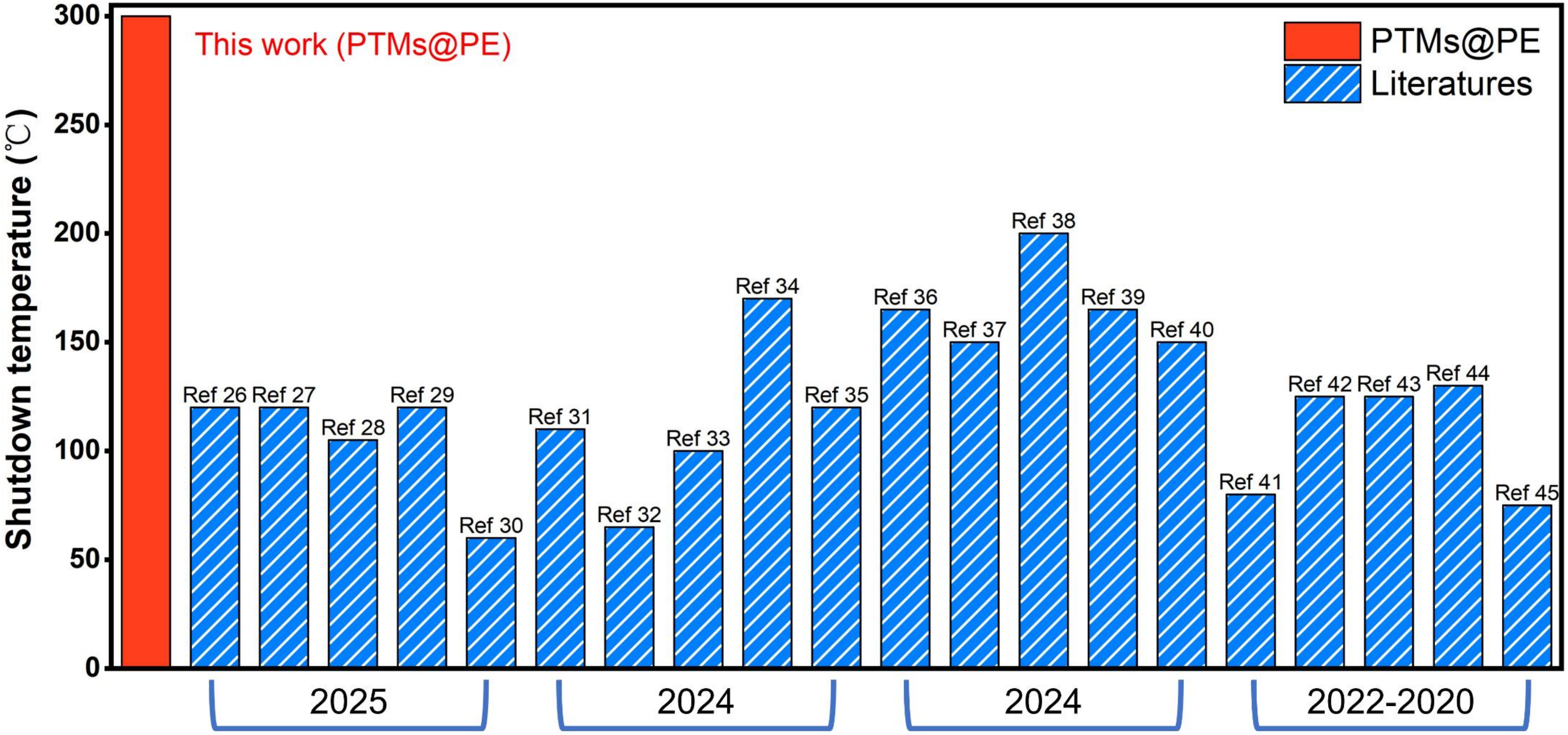

Figure S1 the shutdown temperature of PTMs@PE separator superior to the developed separator in latest five years

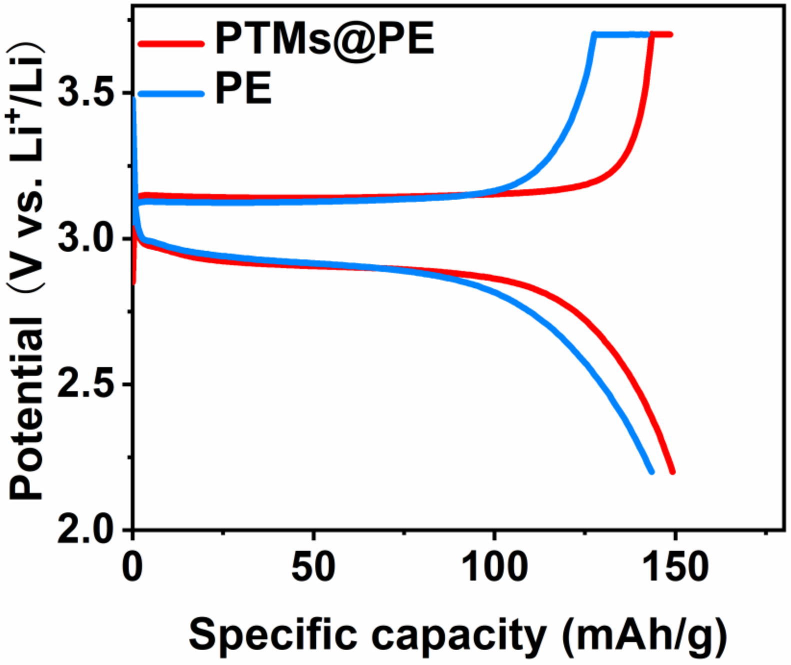

Figure S2 GCD curves（2C）of PE and PTMs@PE separators.

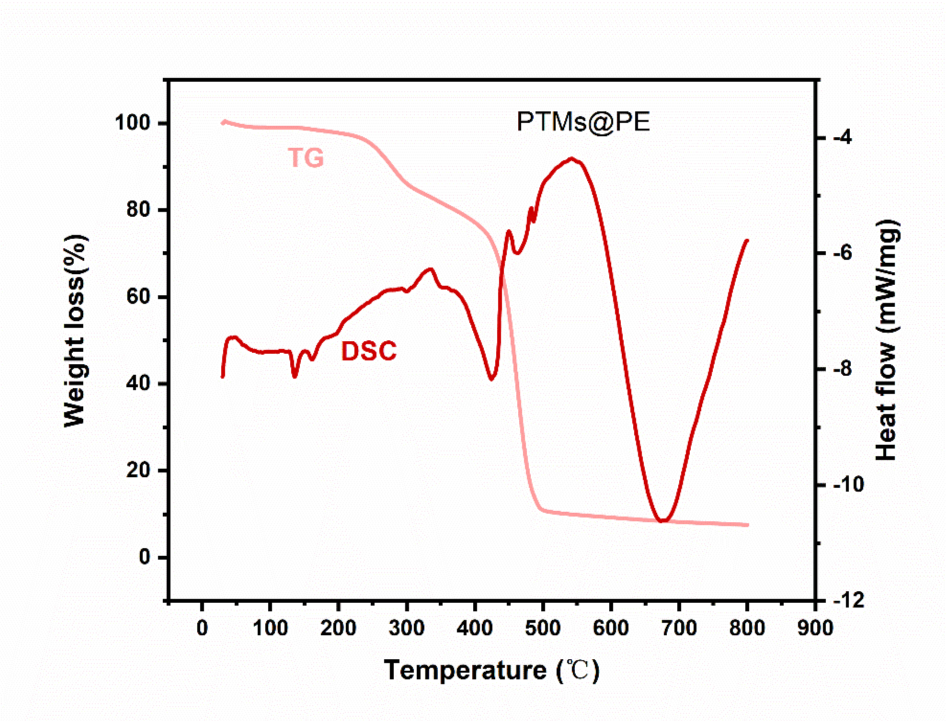

Figure S3 STA curves of PTMs@PE separators.

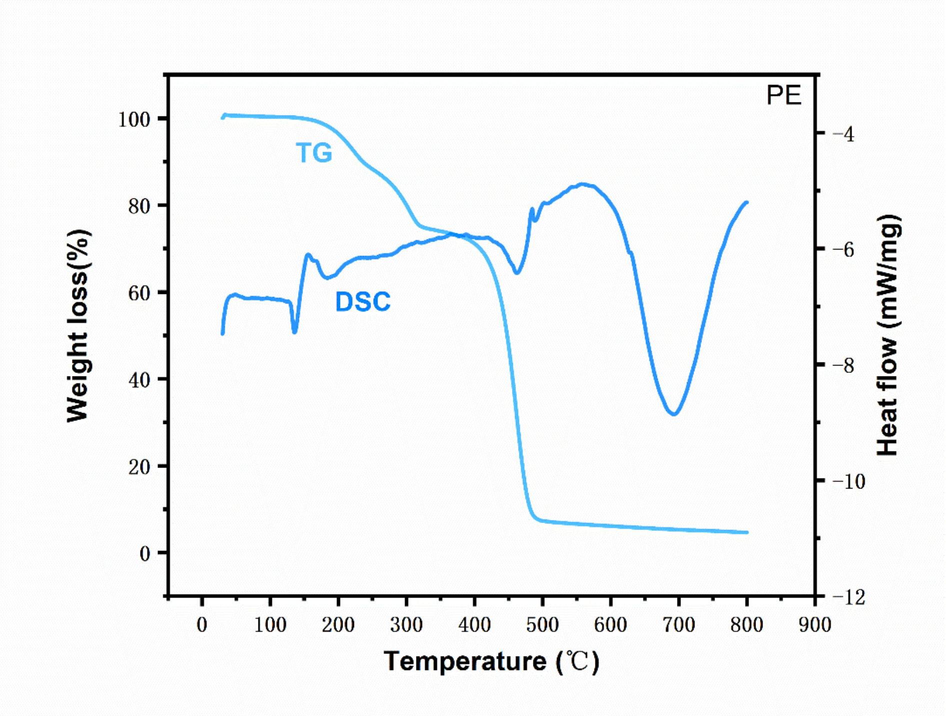

Figure S4 STA curves of PTMs@PE separators.
